# No effects of 1 Hz offline TMS on performance in the stop-signal game

**DOI:** 10.1038/s41598-023-38841-z

**Published:** 2023-07-18

**Authors:** Maximilian A. Friehs, Julia Siodmiak, Michelle C. Donzallaz, Dora Matzke, Ole Numssen, Christian Frings, Gesa Hartwigsen

**Affiliations:** 1 Lise-Meitner Research Group Cognition and Plasticity, Max Planck Institute for Human Cognitive and Brain Sciences, Leipzig, Germany; 2 Department of General Psychology and Methodology, Trier University, Trier, Germany; 3 School of Psychology, University College Dublin, Dublin, Ireland; 4 Department of Psychology, Psychological Methods Unit, University of Amsterdam, Amsterdam, The Netherlands; 5 University of Gdansk, Gdańsk, Poland; 6 Psychology of Conflict Risk and Safety, University of Twente, Enschede, The Netherlands; 7 Wilhelm Wundt Institute for Psychology, Leipzig University, Leipzig, Germany

**Keywords:** Cognitive neuroscience, Human behaviour

## Abstract

Stopping an already initiated action is crucial for human everyday behavior and empirical evidence points toward the prefrontal cortex playing a key role in response inhibition. Two regions that have been consistently implicated in response inhibition are the right inferior frontal gyrus (IFG) and the more superior region of the dorsolateral prefrontal cortex (DLPFC). The present study investigated the effect of offline 1 Hz transcranial magnetic stimulation (TMS) over the right IFG and DLPFC on performance in a gamified stop-signal task (SSG). We hypothesized that perturbing each area would decrease performance in the SSG, albeit with a quantitative difference in the performance decrease after stimulation. After offline TMS, functional short-term reorganization is possible, and the domain-general area (i.e., the right DLPFC) might be able to compensate for the perturbation of the domain-specific area (i.e., the right IFG). Results showed that 1 Hz offline TMS over the right DLPFC and the right IFG at 110% intensity of the resting motor threshold had no effect on performance in the SSG. In fact, evidence in favor of the null hypothesis was found. One intriguing interpretation of this result is that within-network compensation was triggered, canceling out the potential TMS effects as has been suggested in recent theorizing on TMS effects, although the presented results do not unambiguously identify such compensatory mechanisms. Future studies may result in further support for this hypothesis, which is especially important when studying reactive response in complex environments.

## Introduction

Stopping before a crosswalk when the traffic light suddenly changes to red or not telling the joke you planned to tell after realizing it's not appropriate are a few of the many examples of effective response inhibition in everyday situations. Inhibitory control is one part of cognitive control that, by effective suppression of behavior after initiation, allows one to navigate through a constantly changing environment and is fundamental for daily well-being and sometimes survival. Inhibitory control is generally conceptualized as a capacity to suppress one's own unwanted actions, thoughts, or feelings after recognizing them as currently undesired. This mechanism is key to select appropriate responses, suppress irrelevant stimuli and achieve relevant goals^1^. Deficits in inhibitory control are common in neurological patients diagnosed with Alzheimer's, Parkinson’s or frontal lobe lesions^2–4^, as well as patients with depression, mania, obsessive compulsive disorder (OCD) and patients fighting obesity^5–7^. Although attention-deficit hyperactivity disorder (ADHD) and schizophrenia are frequently associated with decreased inhibitory control, these effects may also be due to impairments in early attentional processes^8,9^. Impaired inhibitory control may lead to impulsive behavior and intrusive, ruminating thoughts and is considered a factor increasing the likelihood of addiction and acts of aggression. There are many ways of measuring inhibitory control but the stop signal task (SST) is arguably one of the most widely used paradigms to study reactive inhibitory control in the laboratory^10^.

### Studying inhibitory control with the stop signal task

In the standard SST, participants are required to press a specific button whenever a cue (an arrow pointing left or right) is presented on a screen unless a “stop” cue is presented after the first one, indicating that participants must refrain from any reaction, hence inhibiting it. They are instructed to respond as quickly as possible and the time difference between “go” and “stop” cues, called Signal-stop-delay (SSD), differs making it easier or harder to withhold the response. Performance in this task is highly variable among the healthy population and often reaches abnormally low scores in patients with poor action inhibition. To quantify individual performance, both speed and accuracy are considered and the time to successfully stop the initiated action is calculated based on participants’ performance estimating the stop-signal reaction Time (SSRT)^11,12^. As the Stop-Signal task is based on an independent race model^13^, in which the cognitive process of “go” reaction and “stop” reaction are two separate processes, whichever process reaches completion first, determines the resulting behavior. Accordingly, earlier completion of the go process results in failure to inhibit a response while earlier completion of the “stop” process leads to successful response inhibition. The SSRT reflects the latency of the stop process, i.e. how quickly a response can be stopped.

However, while the SST is commonly used in lab-based research, it also has its drawbacks and can be monotonous for participants. Recently, several research teams have developed gamified stop-signal tasks^14,15^, which do not change the underlying architecture of the SST but yield more enjoyable experiences for participants. Further, these gamified tasks provide a more engaging environment which creates a more captivating setting that may aid in collecting data from populations with a lower attention span such as children or groups of patients with concentration or attention deficits^16–19^.

### The neurophysiological underpinnings of response inhibition

Neuroimaging studies in the past have identified key brain areas and circuits for executive processes. One of the largest brain areas associated with cognitive control is the prefrontal cortex (PFC), postulated as a center of control processes already by Luria in 1962 (for a recent version see Luria, 2012^20^). In particular, response inhibition processes are associated with activity in the dorsolateral prefrontal cortex (DLPFC) and the right inferior frontal gyrus (IFG)^21–24^. Hinting at their general relevance in the cognitive control network, both DLPFC and IFG are also implicated in other complex cognitive processes such as error detection and conflict resolution^25^.

The DLPFC is further associated with working memory as well as selective attention^26^ and can be activated during emotional regulation processes^27,28^ to exert top-down influence on emotional reactivity^29^. It is also involved in conscious decision making, outcome prediction and, of course, inhibition^30^. Lesion studies show that DLPFC damage leads to less efficient response inhibition during the stop-signal task^2^. One key assumption is that the prefrontal cortex does not act independently, but rather changes the actions and signals from other areas, and the resulting cognitive control stems from active goal maintenance; a process closely connected to DLPFC activity^24^. The DLPFC may be viewed as a more domain general processing area involved in a multitude of cognitive processes, such as working memory and emotional or memory suppression, as evidenced by overlapping activation within the area in several different tasks^23,26,31^. Further, DLPFC activity has been shown to be, for example, connected with food-related impulsivity and risk-taking behavior further indicating that the right DLPFC is involved in executive functioning on a domain general level^32,33^*.*

Studies investigating right IFG activity consistently implicate the area in response inhibition tasks^34,35^. Further, lesion studies provide additional evidence for a direct link of the right IFG and response inhibition^36^. However, some studies suggest that the rIFG is not only active during response inhibition, but also during target detection tasks^35,37^. This fits recent evidence from a lesion study suggesting that the rIFG is partially responsible for detecting salient signals (such as stop-signals), leading to the triggering of an inhibitory control process^38^. The rIFG is connected with the pre-SMA, an important area for voluntary action inhibition. Specifically, it has been proposed that the rIFG acts as ‘emergency break’ when the need to stop an action arises^21,39–41^. Thus, the right IFG can be conceptualized as a domain specific processing area, responsible for motor response inhibition^21,42^.

The involvement of specific brain areas in the response inhibition process can be directly probed with non-invasive brain stimulation procedures. Although some previous studies have used transcranial magnetic stimulation (TMS) over the right PFC to modulate response inhibition in the SST, the results are heterogeneous and the interaction of the specific subregions within the right PFC (i.e., rDLPFC and rIFG) and their individual contributions to the response inhibition process remains unclear. Specifically, stimulation over the right IFG can lead to a performance deficit in the SST^43–45^ or no effect at all^46^. Even fewer studies investigated TMS effects over the right DLPFC on response inhibition in the SST and the results show no effect of TMS on the response inhibition process even though the prevailing theory suggests that perturbation of that area should impact performance^22,44,47^.

## The present study

The goal of the present study was to investigate the impact of offline TMS (i.e., applied before task processing) over the right IFG and DLPFC on performance in a gamified stop-signal task. The stop-signal game (SSG) is a recently developed and validated adaptation of the SST, in which participants have to navigate through an enchanted forest^14^. The task functions analogous to the SST and is characterized by increased visual demands due to the everchanging 3D images on screen. Importantly, previous research demonstrated that performance between a standard SST and the SSG is comparable, while the SSG is considered more enjoyable for participants^14^. In this study, participants underwent three experimental sessions with either a real stimulation over the IFG or DLPFC or sham TMS. We hypothesized that perturbation of either area would decrease performance in the SSG. However, we expected a quantitative difference in the performance decrease after stimulation. After offline TMS, functional reorganization is possible and the domain-general area (i.e., the right DLPFC) might be able to compensate for the perturbation of the domain-specific area (i.e., the right IFG). Put differently, the effects of TMS on SSG performance were hypothesized to be smaller in the rIFG stimulation condition than in the rDLPFC stimulation condition.

## Results

Overall, performance was in the expected range, and comparable to previous studies utilizing a similar gamified task version^14,15,48–50^. For details see Table 1.

**Table 1:** Descriptive performance data depending on the TMS condition. Standard deviations shown in brackets. SSRT, SSD and Correct Go-RT are shown in milliseconds and overall accuracy in percent.

	rDLPFC	rIFG	Sham
SSRT	453 (57)	456 (50)	458 (52)
SSD	520 (233)	500 (191)	483 (185)
Correct Go-RT	999 (215)	974 (176)	964 (156)
p(response|inhibition)	0.48 (0.027)	0.47 (0.021)	0.48 (0.022)
Overall go-accuracy	0.99 (0.019)	98 (0.036)	0.98 (0.022)

### SSRT

**Figure 1:**
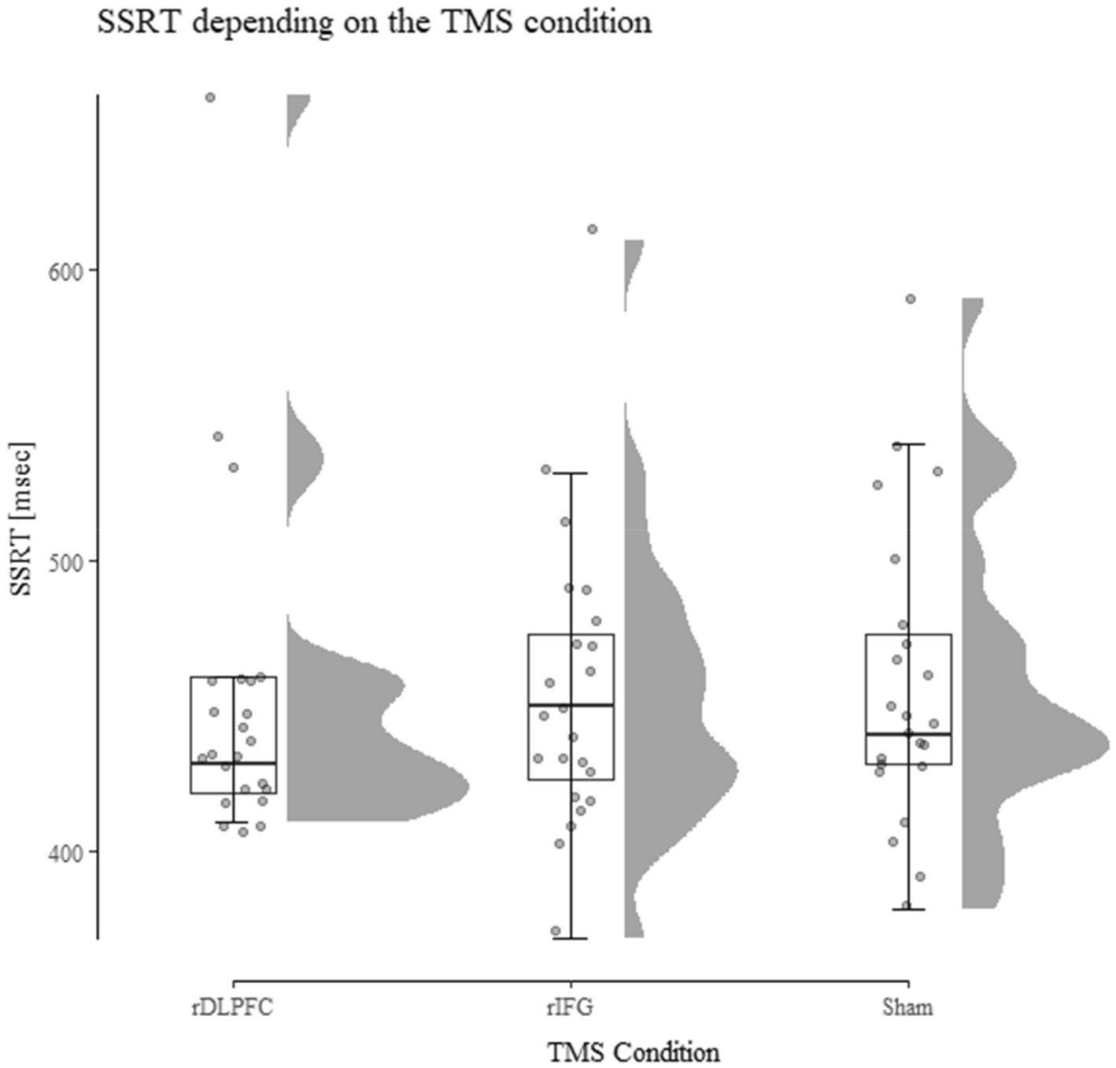
SSRT by TMS Condition as computed by the integration method. Results show no difference in SSRT depending on the TMS Condition. SSRT on the y-axis is displayed in msec. Descriptively, the variance in the rDLPFC condition is lower compared to the other conditions.

Results of a 6 (Order) × 3 (TMS Condition: rDLPFC vs. rIFG vs. sham) repeated measures ANOVA showed neither a significant main effect of TMS Condition (*F*(2, 34) = 0.186, *p* = 0.83), nor a main effect of Order (*F*(5, 17) = 1.4, *p* = 0.27) nor an interaction (*F*(10, 34) = 0.432, *p* = 0.93). For a visual representation see Fig. 1. We included order as a between-subjects factor in the analysis to control for potential sequence effects. Further, complementary Bayesian analysis revealed a BF01 = 6.94 in favor of the null-hypothesis with regards to the main effect of TMS Condition. We used a Cauchy prior distribution with *r* = 0.707 since this prior reflects the range of most psychological effects^51^. Bayesian analysis was done with JASP Version 0.17.1 (JASP Team 2023).

### Error rates

A 6 (Order) × 3 (TMS Condition: rDLPFC vs. rIFG vs. sham) repeated measures ANOVA revealed no main effect of TMS Condition (*F*(2, 34) = 0.13, *p* = 0.88), and no main effect of Order (*F*(5, 17) = 0.32, *p* = 0.89) or an interaction (*F*(10, 34) = 1.52, *p* = 0.17). Further, complementary Bayesian analysis revealed a BF01 = 7.20 in favor of the null-hypothesis with regards to the main effect TMS Condition.

### SSD

A 6 (Order) × 3 (TMS Condition: rDLPFC vs. rIFG vs. sham) repeated measures ANOVA revealed neither a main effect of TMS Condition (*F*(2, 34) = 1.88, *p* = 0.17), nor a main effect of Order (*F*(5, 17) = 2.05, *p* = 0.12) nor an interaction (*F*(10, 34) = 0.92, *p* = 0.53). Further, complementary Bayesian analysis reveals a BF01 = 2.18 in favor of the null-hypothesis with regards to the main effect TMS condition.

### Go RT

Results of a 6 (Order) × 3 (TMS Condition: rDLPFC vs. rIFG vs. sham) repeated measures ANOVA showed neither significant main effect of TMS Condition (*F*(2, 34) = 2.13, *p* = 0.13), nor a main effect of Order (*F*(5, 17) = 1.97, *p* = 0.34) nor an interaction (*F*(10, 34) = 0.87, *p* = 0.57). Further, complementary Bayesian analysis reveals a BF01 = 2.39 in favor of the null-hypothesis with regards to the main effect TMS Condition.

### Bayesian modeling results

The population-level parameters from the hierarchical model were estimated as location and scale parameters but transformed into means and standard deviations before interpreting them.The go and stop trigger failure parameters were estimated on the real line and were transformed back to the probability scale using a bivariate inverse probit transformation. We used the means of the transformed posterior distributions as point estimates. To quantify posterior uncertainty, we computed 95% credible intervals (i.e., the 2.5th and 97.5th percentile of the posterior samples). Table 2 shows the posterior means and corresponding credible intervals of the population-level model parameters across conditions. To compute the posterior distribution of mean Go-RT and SSRT, we summed up the independent MCMC samples for the respective \mu and \tau parameters on each iteration and collapsed them across chains.

**Table 2:** Posterior means of model parameters and their 95% credible intervals. SSRT and Go-RT are shown in seconds. Go and Trigger failures, P(GF) and P(TF), are shown as probabilities.

	rDLPFC	rIFG	Sham
${\mu }_{go}$	0.897 [0.799, 0.992]	0.872 [0.799, 0.946]	0.868 [0.803, 0.935]
${\sigma }_{go}$	0.097 [0.075, 0.123]	0.094 [0.074, 0.117]	0.099 [0.076, 0.128]
${\tau }_{go}$	0.102 [0.083, 0.123]	0.104 [0.082, 0.127]	0.099 [0.078, 0.121]
Correct Go-RT ${\mu }_{go}+{\tau }_{go}$	0.999 [0.901, 1.10]	0.976 [0.9, 1.06]	0.967 [0.897, 1.04]
${\mu }_{stop}$	0.415 [0.396, 0.435]	0.417 [0.402, 0.433]	0.411 [0.397, 0.427]
${\sigma }_{stop}$	0.038 [0.027, 0.051]	0.036 [0.025, 0.051]	0.036 [0.026, 0.050]
${\tau }_{stop}$	0.036 [0.025, 0.051]	0.043 [0.03, 0.06]	0.052 [0.036, 0.073]
SSRT ${\mu }_{stop}+{\tau }_{stop}$	0.452 [0.429, 0.475]	0.461 [0.44, 0.483]	0.463 [0.442, 0.488]
P(GF)	0.012 [0.005, 0.028]	0.014 [0.006, 0.032]	0.018 [0.006, 0.043]
P(TF)	0.056 [0.024, 0.118]	0.047 [0.023, 0.085]	0.059 [0.037, 0.092]

**Figure 2:**
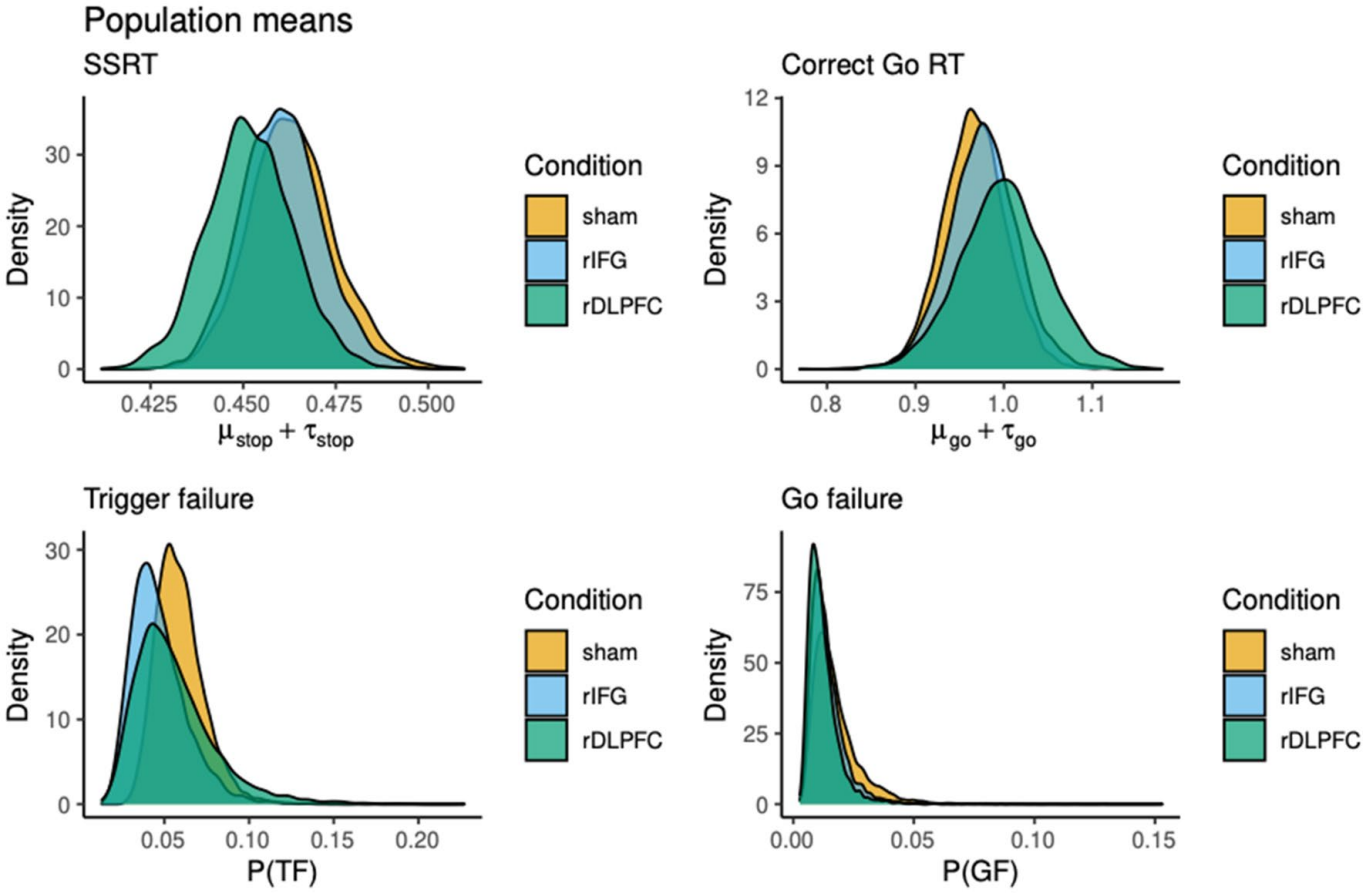
Posterior distributions of the population-level mean parameters across conditions. *SSRT* stop-signal reactiontime, *RT* Response time, *P(TF)* stop trigger failure probability, *P(GF)* go trigger failure probability. SSRT and Correct Go RT are on the millisecod scale, stop and go trigger failures are on the probability scale.

Parameter differences between conditions were explored using pairwise Bayesian p-values (BP). BPs denote the proportion of posterior samples that is greater than the posterior samples in the comparison condition. If a BP is close to 1 or 0, the posterior distribution of the comparison condition is shifted to lower or higher values, respectively, which suggests that the parameter of interest differs between conditions. The pairwise BPs for SSRT ranged from 0.239 to 0.426, for correct Go-RT, from 0.285 to 0.42, for trigger failures from 0.26 to 0.4, and for go failures from 0.304 to 0.407, overall indicating greatly overlapping posterior distributions and hence no reliable differences (see Fig. 2).

## Discussion

In this study, we probed the functional relevance of the right IFG and right DLPFC for reactive response inhibition. We observed no effect of 1 Hz offline rTMS over either brain area on performance. Importantly, there was no TMS effect with neither standard frequentist methods, nor with Bayesian modeling analyses. In fact, SSRT estimates—the most important performance measure—differed on average only by a few milliseconds between conditions and the posterior distributions showed a large degree of overlap. Especially the mean SSRT posterior distributions of the sham and the rIFG stimulation conditions and the individual SSRTs, as computed by the integration method, look almost identical (see Figs. 1, 2), whereas the posterior distribution for rDLPFC was slightly shifted towards lower SSRT; however that minor shift was far from reliable.

Further, Bayesian modeling revealed no difference between the stimulation conditions with regards to stop or go trigger failure probability,indicating that the present null result in SSRT cannot be attributed to differences in the likelihood of launching the stop and go processes. Recent research provided evidence for a higher degree of trigger failures and a slower SSRT (mainly carried in mu_stop) in rIFG lesion patients^38^. Consequently, it would have been plausible to assume that disruption via TMS to the rIFG would lead to a similar pattern of results. With that being said, it is unclear what exact results pattern would be expected for a successful disruption of the rDLPFC.

Although we expected a performance disruption after stimulation, these results overall fit the heterogeneous results in the literature. For example, while Chambers et al.^44^ found no effect of 15 min of 1 Hz at, on average, 92% distance-adjusted RMT over the right DLPFC (for a similar result see also Upton et al.^47^), they did observe a perturbation after right IFG stimulation (see also Lee et al.^46^). In another study, offline continuous theta burst stimulation yielded a behavioral effect on performance after stimulating the right anterior insula, close to the right IFG, but no effect after right middle frontal gyrus stimulation^22^.

There are several factors that may contribute to the null-effects in the present study besides compensatory mechanisms as well as the heterogeneous results in the literature. First, although TMS is among the more precise NIBS methods, without previous functional MRI it is not possible to ensure that each person is stimulated in exactly the right area and neuronal activity in non-target regions may be modulated instead. Second, not all brain areas make for equally good targets and stimulation spreads to neighboring cortical areas depending on the individuals’ brain anatomy^52^. Third, there are large interindividual differences with regards to TMS- and more generally NIBS-effects on performance^53,54^. Apart from the difference in neuroanatomy, there are also baseline activity differences to be considered and some authors report on a brain state dependent stimulation effects^55,56^. This inter- and even intra-individual variability to TMS may mask potential TMS effects and increase the likelihood of null-results and a non-significant result may not necessarily equate to an absence of effect. Fourth, different TMS protocols impact the type of inference that is possible. Thus for example, while online interference protocols (e.g., 10 Hz rTMS in short bursts during stimulus processing) cause a disruption of an ongoing task, offline inhibition protocols (e.g., 1 Hz TMS or cTBS before task performance) are assumed to decrease cortical excitability via presumably long-term depression of the stimulated synapses^54^. Fifth, given the possibility of short-term cortical reorganization it may be possible that the lack of TMS effect on performance may be due to within-network compensation^54,57^. Put differently, it is possible, or even likely, that 1 Hz TMS over one area led to an up-regulation of another area which helped to compensate for the perturbation. Given the duration of the stimulation and the fact that no TMS was applied during task performance, this compensation effect could have nullified any potential TMS effect. These lines of reasoning and results also fit with recent transcranial direct current stimulation (tDCS) studies^58–60^. However, compensation of TMS-based perturbation is not always possible and compensatory mechanisms may break down when cognitive demands are especially high^61^ or due to their inherent asymmetry. Specifically, domain-general areas may be able to compensate for disturbance in process-specific areas but compensation of domain-general area disruption by domain-specific areas is less likely^57,62–64^.

The ability of the human brain to flexibly reorganize processes to changing situations and task requirements and to compensate for perturbations is astonishing^57^. For example, some research suggests that an upregulation of regions associated with the domain-general network can drive language recovery after lesions to specialized language areas in aphasia patients^63,65^. It has further been suggested that the brain is primed for neuroplasticity after a stroke, which can lead to heightened effectiveness of rehabilitative therapy^66,67^. Thus, the fact that we did not observe a TMS effect on performance may suggest a potential compensatory effect that future studies may probe. There are several factors that may contribute to the null-effects in the present study besides compensatory mechanisms as well as the heterogeneous results in the literature. First, although TMS is among the more precise NIBS methods, without previous functional MRI, it is not possible to ensure that each person is stimulated in exactly the right area and neuronal activity in non-target regions may be modulated instead^54^. Second, not all brain areas are equally suitable targets and stimulation can spread to neighboring cortical areas,; both of which can impact stimulation outcomes^52^. Third, there are large interindividual differences with regards to TMS—and more generally NIBS-effects—on performance^53,54^. Apart from differences in neuroanatomy, baseline performance and activity differences should also be considered, which may lead to a brain-state dependent stimulation effects^55,56^. Such inter- and even intra-individual variability to TMS may mask potential TMS effects and increase the likelihood of null-results. Consequently, a non-significant result may not necessarily equate to the absence of an effect. However, baseline differences are usually ignored in TMS studies^68^. With that being said, although Bayesian analyses cannot overcome these challenges, they can provide evidence for the null hypothesis. Accordingly, our complementary Bayesian analysis revealed moderate evidence in favor of the null (for Bayes Factor interpretation see Lee and Wagenmakers, 2014^69^). Fourth, different TMS protocols may differentially impact task performance. For example, while online interference protocols (e.g., 10 Hz rTMS in short bursts during stimulus processing) can results in a disruption of ongoing task performance, offline inhibition protocols (e.g., 1 Hz TMS or cTBS before task performance) are assumed to decrease cortical excitability, presumably via long-term depression-like effects on the stimulated synapses^54^. Fifth, given the possibility of short-term cortical reorganization, it is possible that the lack of a clear TMS effect on performance may be due to within-network compensation^54,57^.

There are several possible avenues for future research on the prefrontal inhibition network. For example, researchers may utilize a condition-and-perturb protocol to probe compensatory mechanisms within the right prefrontal cortex. Here, a combined offline and online perturbation of both the right IFG and right DLPFC may result in stronger effects relative to the perturbation of each area alone, which would allow testing the compensation theory. Additionally, researchers may aim to increase stimulation focality by targeting individual activation peaks based on functional localizers obtained from fMRI. Recent advances even allow for the concurrent use of TMS and neuroimaging procedures^70,71^.

Another limitation of the current study is the specific sham condition that was used. To reiterate, the coil was placed on a participant’s head and tilted away to avoid effective cortical stimulation during TMS. A more ideal sham procedure would have been to place the coil directly on the vertex or even stimulate a control site assumed to not be part of the network required for task performance^54,72–74^. In this way, a more realistic sham condition could have mimicked the somatosensory side effects of TMS. However, while this procedural change would have generally improved the study design, it is unlikely that an active sham condition would have turned the observed null findings into positive effects. Notably, by more closely mimicking the somatosensory effects an active sham condition may slightly reduce the likelihood of finding an active TMS effect in comparison by creating a placebo effect^72^. However, although we cannot fully rule out this possibility, the present study employed an offline TMS protocol and any somatosensory effect of an active TMS application would have most likely faded and not affected the task after its cessation.

## Conclusion

In conclusion, 1 Hz offline TMS over the right DLPFC and the right IFG at 110% RMT had no effect on performance in a gamified SST. In fact, evidence in favor of the null hypothesis was found. However it needs to be noted that the BEESTS model cannot prove the null, and evidence for the null hypothesis is based on SSRT estimates via the integration method.. One theoretically intriguing interpretation of this result is that within-network compensation was triggered canceling out the potential TMS effects as has been suggested in recent theorizing on TMS effects.

## Methods

### Sample

27 participants were recruited via the database of Max Planck Institute for Human Cognitive and Brain Sciences, Leipzig, Germany. Written informed consent was obtained from each subject prior to the experiment. The study was approved by the local ethics committee of the Medical Faculty at Leipzig University. All participants were healthy right-handed volunteers aged between 18 and 40, had normal or corrected-to-normal vision, no cardiovascular, neurological or psychiatric disorders or metal implants. Participants were informed about procedures but were blinded regarding the different TMS conditions. Based on the data reduction criteria (see below) the final sample consisted of 23 participants (14 female, mean age = 30.17, SD = 5.4). For sample size estimations and power analysis, we assumed an effect of Cohen’s f = 0.4 for a TMS effect on SSRT, a medium sized correlation between measures of *r* = 0.5 and a Type I and Type II error of 0.05 (using GPower^75^). This calculation resulted in a sample of at least 18 participants. Further, this sample size is in line with previous research investigating stop-signal task performance. For example, some studies similar to the present study in terms of design employed samples sized between 8 and 24^22,44,46,47^.

### Design

Each participant underwent three experimental sessions that varied in TMS site (rIFG vs. rDLPFC vs. sham). After preparation and a practice block, each participant performed 450 experimental trials of the SSG per session, split into nine blocks. Each session lasted approximately 1.5–2.5 h. The individual resting motor threshold was determined in the first session. Sessions were separated by at least 7 days to prevent carry-over effects. The order of sessions was counterbalanced across participants to the best possible degree. Our design resulted in six possible stimulation sequences.

### Transcranial magnetic stimulation

We applied 30 min of neuronavigated 1 Hz TMS (TMS Navigator, Localite, Sankt Augustin, Germany) prior to the SSG (i.e., offline TMS). 30 min of 1 Hz TMS has been shown to decrease regional cerebral glucose metabolic rates as evidenced by PET scans^76^ and influenced various cognitive processes such as action reprogramming and memory formation^77–79^. Moreover, effects appear to be intensity-dependent, with higher intensities leading to a stronger effect^80,81^. The stimulation was based on co-registered individual T1-weighted MR images to navigate the TMS coil and maintain its exact location and orientation throughout all sessions. T1-weighted images were taken from the in-house database or acquired at a 3-Tesla MRI (Prisma, Siemens Healthcare, Germany) using a magnetization prepared rapid gradient echo (MPRAGE) sequence in sagittal orientation (inversion time = 650 ms, repetition time = 300 ms, flip angle = 10°, field of view = 256 mm × 240 mm, voxel-size = 1 mm × 1 mm × 1.5 mm). TMS was performed using the average Montreal Neurological Institute (MNI) coordinates for the rIFG (x = 50, y = 19, z = 16) and rDLPFC (x = 40, y = 32, z = 36) based on previous studies and meta-analysis^22,34,43,44,46,82–86^. Note that the exact labeling of these prefrontal areas is inconsistent across studies and the reported activation peaks and stimulation sites are heterogeneous. Individual stimulation targets for rIFG and rDLPFC were obtained by using the inversed normalization procedure in SPM 8 (Welcome Trust Center for Neuroimaging, University College London, UK) to transform the MNI coordinates to individual space. Sham TMS was applied over the vertex, which was determined as the midpoint between the lines connecting the nasion and inion and tragi of the left and right ear. At the beginning of each experimental session, participants were co-registered to their structural T1. Individual resting motor thresholds (rMTs) were determined in the first session^61,87^. Stimulation intensity was set to 110% of the participant’s rMT^47^. The coil was placed tangentially on the head with the handle pointing at 45° to the sagittal plane for both active TMS conditions. A figure-of-eight coil (CB-60; double 60 mm) connected to a MagPro X100 stimulator (MagVenture, Denmark) was used, and the overall application of TMS pulses was within recommended safety limits^88,89^. During the individual session, the coil was held in place by the experimenter. Accurate coil positioning and maintenance were achieved with a neuronavigation system, which was placed behind the participant but visible to the experimenter. Participants were asked to lean against a custom-made headrest with the back of their head and avoid movements during the experiment. All participants tolerated this procedure and completed the whole experiment. Note that in case of discomfort we reduced the stimulation intensity by 1–2%. Specifically, in 8 out of 46 active sessions a reduction of at most 2% maximum stimulator output (MSO) was needed. Further, 3 participants (i.e. 6 active sessions) required a reduction in both active stimulation conditions. Note that the average stimulation intensity was 110.48% (SD = 1.05) for the rDLPFC and 110.49% (SD = 1.01) for the rIFG. For the sham condition, the coil was oriented parallel to the sagittal plane and placed across the vertex. Importantly, the coil was tilted away from the head in the sham condition to avoid any effective stimulation of the underlying brain tissue; in this case only the rim of the coil touched the participants’ head.

**Figure 3:**
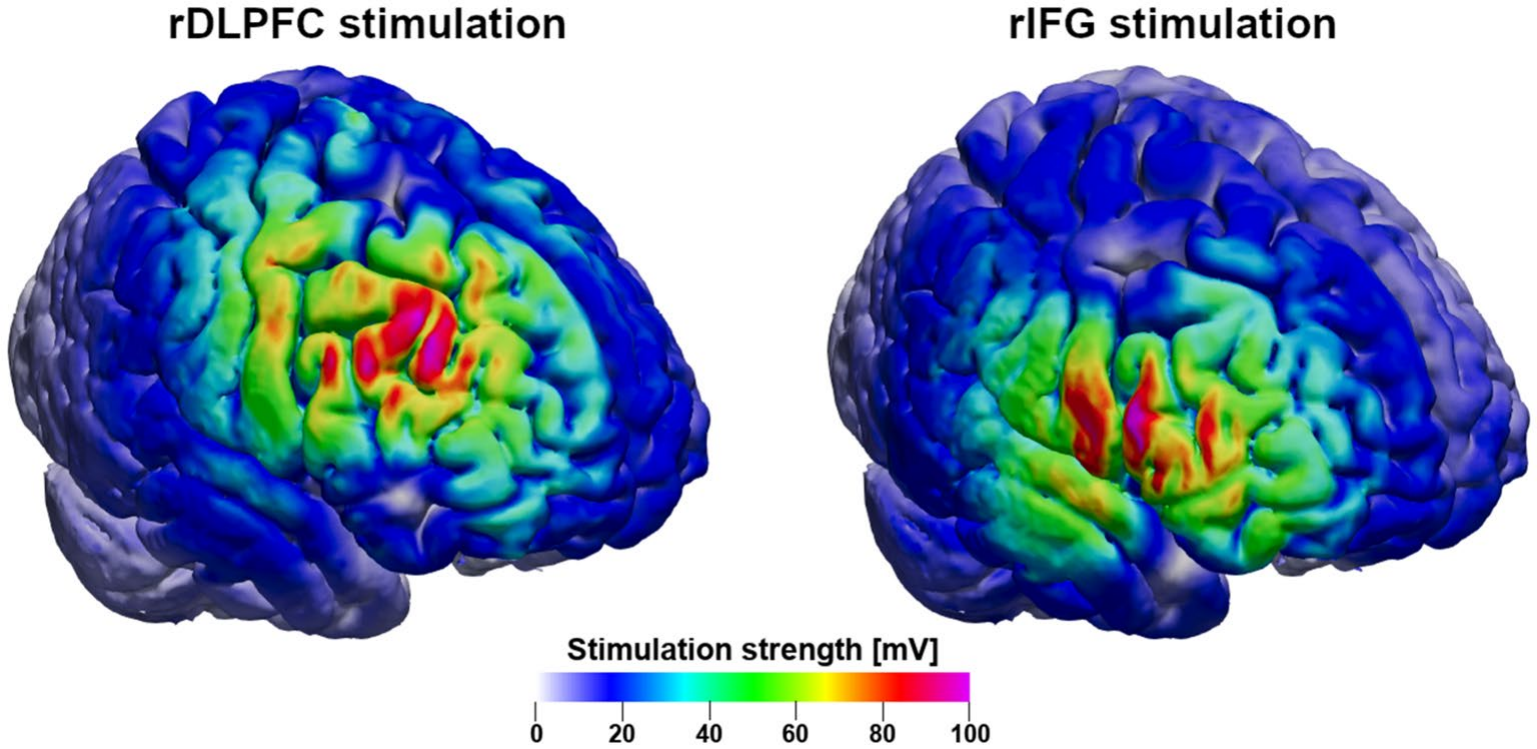
Both cortical sites were effectively targeted with TMS. The stimulation exposed the cortical target (left: rDLPFC; right: rIFG) to significant electrical stimulation. Critically, the off-target regions, including the alternative target, were stimulated significantly less (green areas) than the cortical target. Color: overall stimulation strength |E|, shown on a grey matter surface for an exemplary subject. Simulation was performed with realistic parameters, i.e., 110% rMT intensity and CB-60 coil model.

To validate stimulation conditions, we performed post-hoc electrical field simulations of the TMS-induced electric fields for the rDLPFC and rIFG conditions. We used SimNIBS v3.2.6 to construct high-resolution geometric head models^90,91^ from individual MRI data (Puonti et al., 2016), employing SPM12 and CAT12^92^ and to estimate individual field exposures via the finite element method (FEM). The final head models were composed of ~ 1.7 × 10^6^ nodes and ~ 9.5 × 10^6^ tetrahedra. T1 images and, if available, T2 images were used for segmenting the following tissues: scalp, skull, grey matter (GM), white matter (WM), cerebrospinal fluid (CSF), and eyes. Standard conductivity values for the tissue types were used. We utilized the coil positions saved by the neuronavigation software to define the position and orientation of the TMS coil for the field simulations^93,94^. We visually assessed the individual field simulations to assure effective stimulation of the cortical targets and differential stimulation patterns across the TMS conditions. Figure 3 shows the e-field simulations of an exemplary subject.

### Task

**Figure 4:**
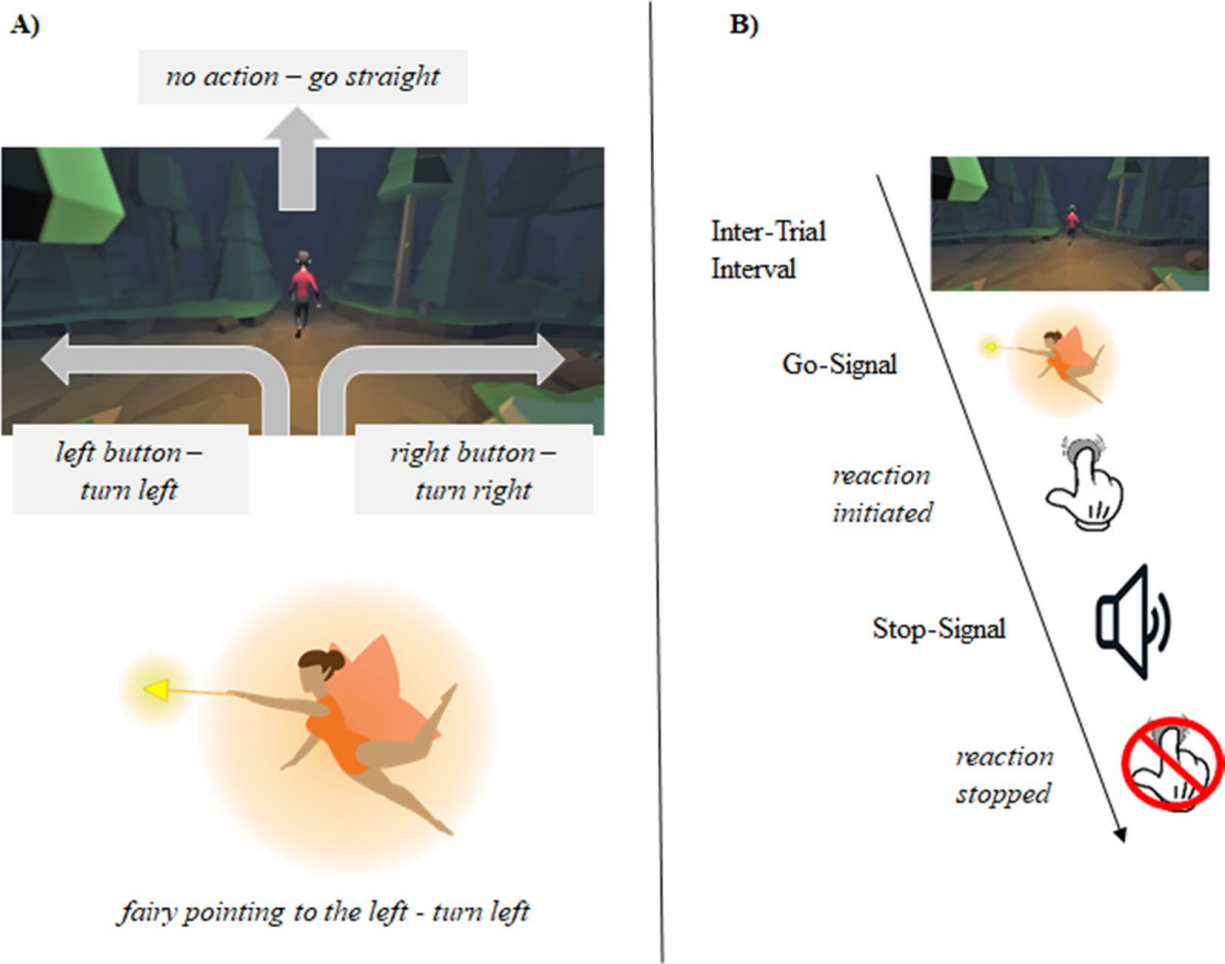
Visualization of the Stop-Signal Game. (**A**) The picture on top shows the basic layout of the SSG environment. The character runs through a procedurally generated forest and has the option to turn left or right at each upcoming intersection. If no button is pressed and no direction is chosen, the character will continue to run straight ahead. The picture on the bottom showsa representation of the basic go-stimulus that is used in the SSG. The fairy appears at each intersection and either points to the left or to the right. (**B**) A prototypical trial in the SSG. During the inter-trial interval the avatar will move through the forest on its own. At an intersection a go-signal in the form of a fairy would appear to indicate the direction the participant should press on the keyboard. After a variable delay, an auditory stop-signal may be presented on a subsection of trials after which a participant should withhold their already initiated action.

Response inhibition was measured using a validated stop-signal game that is conceptually identical to the ordinary SST^14,48^. The SSG is a 3D game in the infinite runner genre, in which the participant has to navigate a character through an enchanted forest. Importantly, the SSG retains measurement validity and exhibits the same properties as the regular SST, apart from an increase in visual complexity and being generally more enjoyable for participants. This is in line with evidence that game-like elements such as simple narratives and consequential choices enhance motivation^95,96^. Mirroring the ordinary SST exactly, but enhancing ecological validity through visual complexity, the SSG required the participants to react to a visual stimulus (i.e., left or right pointing fairy sprite on the screen); on a random subset of trials an auditory stop-signal (i.e., beep-sound) was presented, which required subjects to withhold their already initiated response. Figure 4 illustrates the SSG. In each trial, the go-stimulus was presented for a maximum of 2000 ms or until the response. The stop-signal was presented via headphones following a variable delay (the Stop-Signal Delay, SSD). The *SSD* represents the delay between the onset of the go- and the stop-signal and was initially set to 250 ms. The SSD was continuously adjusted with a staircase procedure to obtain a probability of responding of 50%. After the reaction was successfully stopped (i.e., button press was inhibited), the SSD was increased by 50 ms, whereas when the participants did not stop successfully, the SSD was decreased by 50 ms. The inter-trial interval was set to a random value between 500 and 1500 ms. Several different performance measures were logged and calculated, including the SSD and the probability of making a (wrong) response when a stop-signal was presented (*p(response|signal)*). Furthermore, two variables that are directly related to accuracy were logged: first, the number of *omission errors* (reflecting the probability of missed responses on go-trials) and second, the number of *commission errors* (reflecting the probability of an incorrect response on go-trials). Additionally, we logged three RT variables; *correct go RT* reflects the speed of correct responses on trials without a stop signal, *incorrect go RT* reflects the response time of wrong go responses (i.e., pressing left when a right turn would have been required or vice versa) and *signal response RT*, which indicates the latency of the incorrectly executed response on stop-signal trials. Furthermore, the probability of a *correct inhibition* (i.e., the likelihood of inhibiting an already initiated action) was recorded for each participant. Most importantly, the stop-signal reaction time (SSRT) could be calculated based on a participant’s performance. The estimation of the SSRT was based on the integration method with replacement of omissions as well as on hierarchical Bayesian parametric modeling^8,10,97^.

### Data reduction

Only participants with three valid datasets (i.e., participated in all three sessions and provided analysable data) were considered for the final analysis. Thus, 3 participants with incomplete datasets were excluded at this stage. In accordance with the recommendations in the literature^10,12^, we screened participants’ performance data in the SSG to make sure that reactive inhibition is accurately estimated. This included testing for the horse-race model assumption by comparing *signal response RT* (i.e., wrongly executed responses on stop-trial) and go RT. The horse-race model dictates that SSRT can only reliably be estimated if mean signal response RT is faster than mean go RT. Further, we will show that there is a statistical difference between the average go-RT (i.e., RTs of correct responses during go-trials) and the average signal response RT (i.e., RTs of false responses during stop-trials) for each experimental condition. Furthermore, we checked whether participants’ p(response|signal) was lower than 0.25 or higher than 0.75 or if their accuracy on go-trials was an outlier based on the Tukey outlier criterion. Additionally, we screened participants' performance for strategic response behavior, based on which we excluded data from one participant (e.g., nearly uniformly distributed RT data). Furthermore, we removed anticipatory responses (i.e., responses faster than 0.2 s; n = 2).

### Bayesian parametric modeling

The data was modeled using BEESTS, a Bayesian parametric approach for the analysis of stop-signal data^8,97,98^. BEESTS is based on the independent horse race model^99^, which assumes that whether a response is successfully inhibited depends on the relative finishing times of a go and a stop runner. The runners are triggered by the go and stop stimuli, respectively. If, on a given stop-trial, the stop runner finishes first, response inhibition is successful. Conversely, if the go runner wins, a response is executed despite the stop-signal, resulting in a signal-response RT. BEESTS allows estimating the SSRT distribution (i.e., the finishing time distribution of the stop runner) which should be considered in its entirety (as opposed to means only). For example, distribution shapes may differ between experimental conditions even if mean SSRTs are the same. Disregarding the shape may lead to erroneous conclusions^100^. Moreover, BEESTS can estimate the probability of go and stop trigger failures, the inability to start the go and stop runners, respectively, which may bias results^8,101^. For example, if ignored, stop trigger failures may lead to overestimation of SSRTs^102^.

In the BEESTS approach, the finishing times of the go and stop runners are modeled as ex-Gaussian distributions with parameters $\mu$, $\sigma$, and $\tau$^97^. The first two parameters reflect the mean and standard deviation of the Gaussian component and the latter the exponential component, accounting for the long slow tail that is characteristic ofRT distributions. The mean of the finishing time distributions (i.e., mean Go-RT and mean SSRT) is the sum of the $\mu$ and $\tau$ parameters (i.e., ${\mu }_{go}+{\tau }_{go}$ and ${\mu }_{stop}+{\tau }_{stop}$). To account for go and stop runner start deficiencies, we used an augmented version of the standard BEESTS that also estimates the parameters P(TF) and P(GF), the probability of triggering the stop and go runners, respectively^8,101^.

After removing all choice errors on go-trials, the model was estimated separately for each experimental condition. To account for the nested data structure, we used hierarchical modeling^103^. For signal response trials (i.e. stop-signal trials), the accuracy of responses with respect to the go stimulus was not recorded, so it was not possible to distinguish between correct and incorrect choices. It is therefore possible that stop-signal trials with incorrect choice data were included in the analysis. However, given the overall low error rate on go-trials, we did not expect the model parameters to be considerably biased by incorrect signal-response trials. Appendix 1 shows the model parametrization and the weakly informative hyperpriors that restricted the parameters to a plausible range. We used the Dynamic Models of Choice software^100^ in the programming environment R (R Core Team, 2019) to fit the models. We assessed model convergence by visually inspecting the Markov chain Monte Carlo (MCMC) chains and by using univariate and multivariate proportional scale-reduction factors ($\widehat{\mathrm{R}}$ < 1.1)^104,105^. The model fits were evaluated using posterior predictive checks. Overall, they provided a good account of the data (see Appendix 1 for more details regarding the fitting procedure and model assessment).

### Ethics statement

The study was approved by the local ethics committee of the Medical Faculty at Leipzig University (# 440/20-ck). All methods were performed in accordance with the relevant guidelines and regulations. All participants provided written informed consent.

## Supplementary Information

Supplementary Information.

## Data Availability

The data is freely available on OSF https://osf.io/w5sry.
