# Supplementary material for: No effects of 1 Hz offline TMS on performance in the stop-signal game
Source: Sci Rep. 2023 Jul 18;13:11565. doi: 10.1038/s41598-023-38841-z (PMC10354051; doi:10.1038/s41598-023-38841-z)
Supplement: Supplementary file 1 — Supplementary Information. [file 41598_2023_38841_MOESM1_ESM.docx]

**Appendix 1**

**Prior specification**

We assumed that the $\boldsymbol{\mu}$**,** $\boldsymbol{\sigma}$, and $\boldsymbol{\tau}$ parameters of individual i, i = 1, …, 23, come from truncated normal population-level distributions with location L, scale S, and lower and upper bounds as follows:

$$\mu_{go\left( i \right)}\sim{Normal}^{+}\left( M\mu_{go},S\mu_{go} \right)\left[ 0,4 \right],$$

$$\sigma_{go\left( i \right)}\sim{Normal}^{+}\left( M\sigma_{go},S\sigma_{go} \right)\left[ 0,4 \right],$$

$$\tau_{go\left( i \right)}\sim{Normal}^{+}\left( M\tau_{go},S\tau_{go} \right)\left[ 0,4 \right],$$

$$\mu_{stop\left( i \right)}\sim{Normal}^{+}\left( M\mu_{stop},S\mu_{stop} \right)\left[ 0,4 \right],$$

$$\sigma_{stop\left( i \right)}\sim{Normal}^{+}\left( M\sigma_{stop},S\sigma_{stop} \right)\left[ 0,4 \right],$$

$$\tau_{stop\left( i \right)}\sim{Normal}^{+}\left( M\tau_{stop},S\tau_{stop} \right)\left[ 0,4 \right].$$

The go and trigger failures were estimated on the real line using probit transformation and only transformed back to probabilities after estimation:

$${TF}_{\left( i \right)}\sim Normal\left( M_{TF},S_{TF} \right)\left[ -6,6 \right],$$

$${GF}_{\left( i \right)}\sim Normal\left( M_{GF},S_{GF} \right)\left[ -6,6 \right].$$

For the population-level location and scale parameters we used the following hyperpriors (all on the seconds scale):

$$M\mu_{go},M\mu_{stop}\sim{Normal}^{+}(0.5,1)[0,4]$$

$$M\sigma_{go},M\tau_{go}, M\sigma_{stop},M\tau_{stop}\sim{Normal}^{+}(0.2,1)[0,4]$$

$$M_{TF}, M_{GF}\sim Normal\left( -1.5,1 \right)\left[ -6,6 \right],$$

$S\mu_{go},S\sigma_{go}, S\tau_{go}, S\mu_{stop}, S\sigma_{stop}, S\tau_{stop}, S_{TF}, S_{GF}\sim Exponential(1)$*.*

**Model estimation**

To fit the models, we used the Dynamic Models of Choice software (DMC; Heathcote et al., 2019) which uses the Differential Evolution Markov chain Monte Carlo sampler (Braak, 2006) in the statistical programming environment R (R Core Team, 2019). We first fit the models for each participant separately and then used the parameter estimates as start values for the hierarchical model. In the burn-in period, the migration step probability was .05. Afterwards, the sampler performed crossover steps only. We ran 24 chains and used thinning to decrease autocorrelation across the MCMC samples. Specifically, every 10th sample from the joint posterior distribution was kept. We assessed model convergence by visually inspecting the MCMC chains and by using univariate and multivariate proportional scale-reduction factors ($\hat{R}$ < 1.1, Brooks & Gelman, 1998; Gelman & Rubin, 1992).

**Model fit assessment**

The model fits were evaluated using posterior predictive checks based on 500 samples from the posterior predictive distribution. Specifically, we examined the average cumulative distribution functions of Go-RTs and signal response RTs, inhibition functions, and median signal response RT as a function of SSD. Across conditions, the models predicted somewhat steeper inhibition functions than were observed in the data, with some misfit in the outer SSDs (see Figures A2, A4, and A6). Overall, the models provided a good account of the data (see Figures A1-A6).


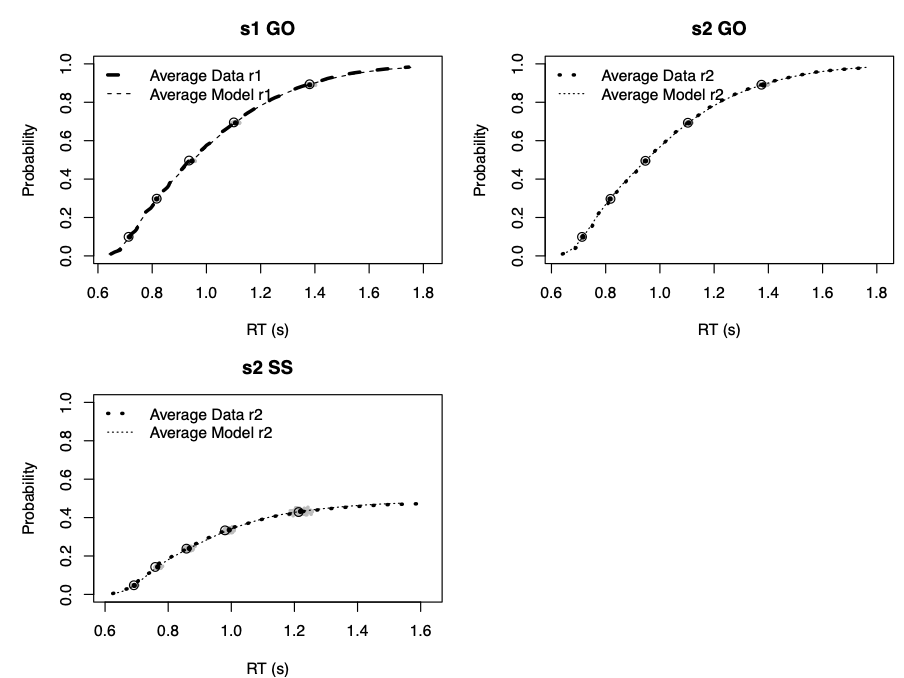


Figure A1. Cumulative distribution functions (CDF) in the rDLPFC condition for the Go-RTs in the upper panels and signal response RTs in the lower panel. The thick lines and dots reflect the observed CDFs and the thin ones the predicted CDFs for responses to the left and right go-stimuli on go trials (i.e., s1 and s2; upper panels) and for responses collapsed across left and right go-stimuli on stop-signal trials (lower panel). All CDFs were averaged across participants and signal response RTs were collapsed across SSDs. The light grey dots denote 500 predicted 10th, 30th, 50th, 70th, and 90th percentiles and the round black circles denote their averages.


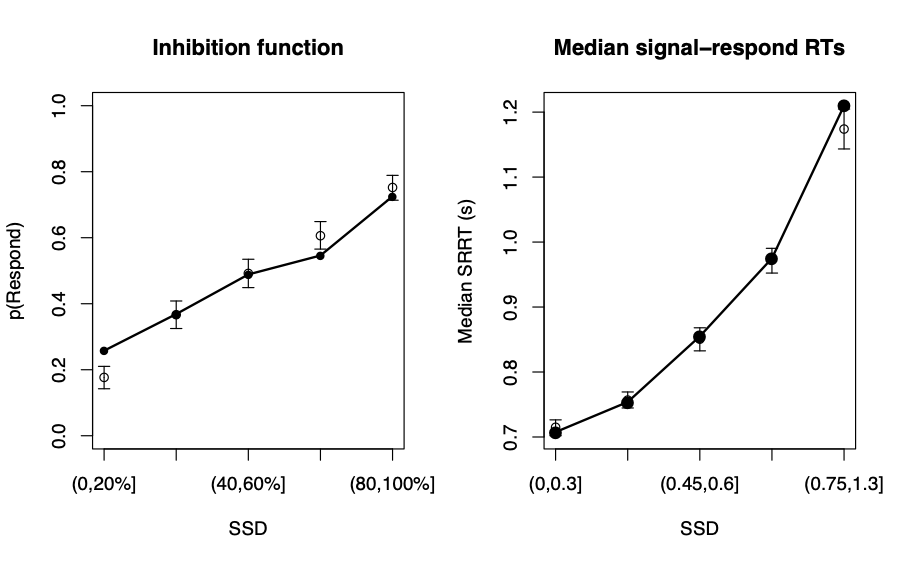


Figure A2. Observed and predicted response proportions (left panel) and median signal response RT (SRRT) as a function of SSDs (right panel) for the rDLPFC condition. The black dots denote the observed data and the circles the median of the 500 samples of the posterior predictive distribution. The error bars denote 95% credible intervals. The SSD categories in the left panel were created by computing the percentiles of the individual SSD distributions and then averaging across individuals. In the right panel, the percentiles were calculated based on pooled individual SSDs.


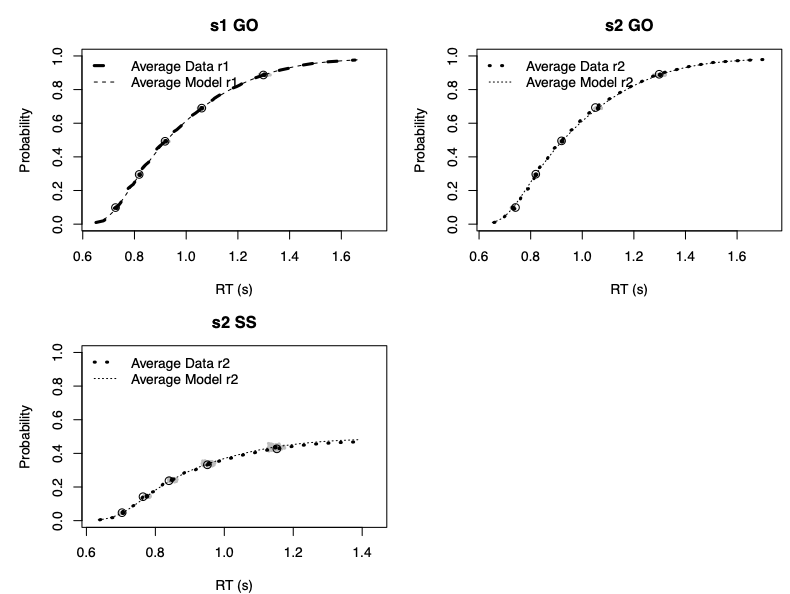


Figure A3. Cumulative distribution functions (CDF) in the rIFG condition for the Go-RTs in the upper panels and signal response RTs in the lower panel. The thick lines and dots reflect the observed CDFs and the thin ones the predicted CDFs for responses to the left and right go-stimuli on go trials (i.e., s1 and s2; upper panels) and for responses collapsed across left and right go-stimuli on stop-signal trials (lower panel). All CDFs were averaged across participants and signal response RTs were collapsed across SSDs. The light grey dots denote 500 predicted 10th, 30th, 50th, 70th, and 90th percentiles and the round black circles denote their averages.


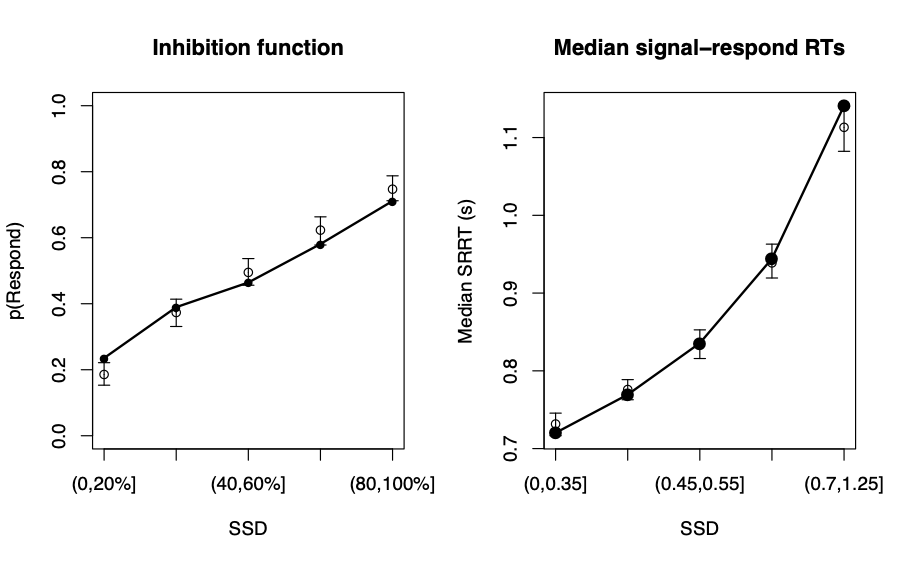


Figure A4. Observed and predicted response proportions (left panel) and median signal response RT (SRRT) as a function of SSDs (right panel) for the rIFG condition. The black dots denote the observed data and the circles the median of the 500 samples of the posterior predictive distribution. The error bars denote 95% credible intervals. The SSD categories in the left panel were created by computing the percentiles of the individual SSD distributions and then averaging across individuals. In the right panel, the percentiles were calculated based on pooled individual SSDs.


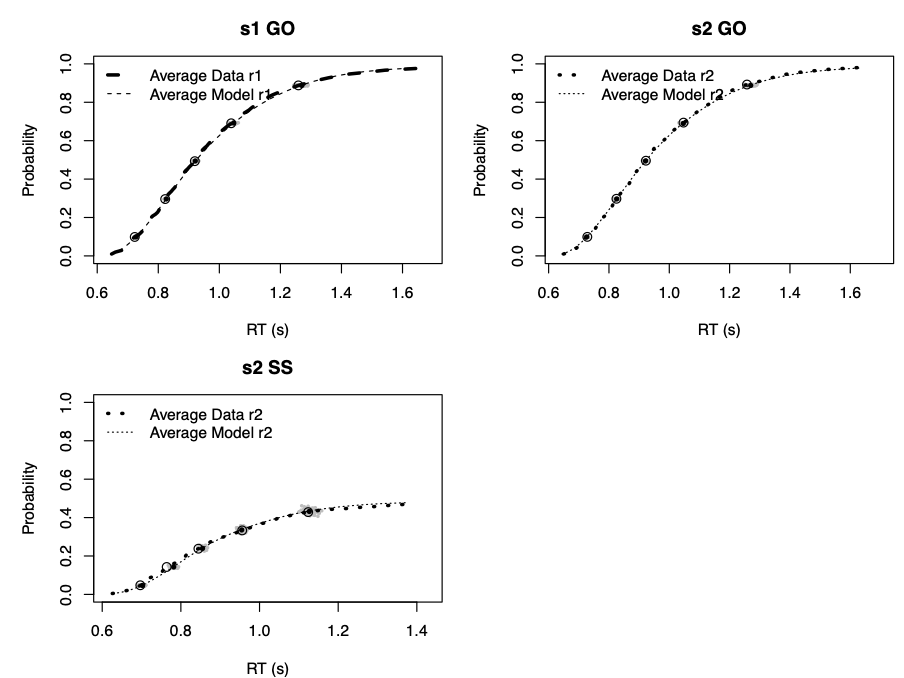


Figure A5. Cumulative distribution functions (CDF) in the sham condition for the Go-RTs in the upper panels and signal response RTs in the lower panel. The thick lines and dots reflect the observed CDFs and the thin ones the predicted CDFs for responses to the left and right go-stimuli on go trials (i.e., s1 and s2; upper panels) and for responses collapsed across left and right go-stimuli on stop-signal trials (lower panel). All CDFs were averaged across participants and signal response RTs were collapsed across SSDs. The light grey dots denote 500 predicted 10th, 30th, 50th, 70th, and 90th percentiles and the round black circles denote their averages.


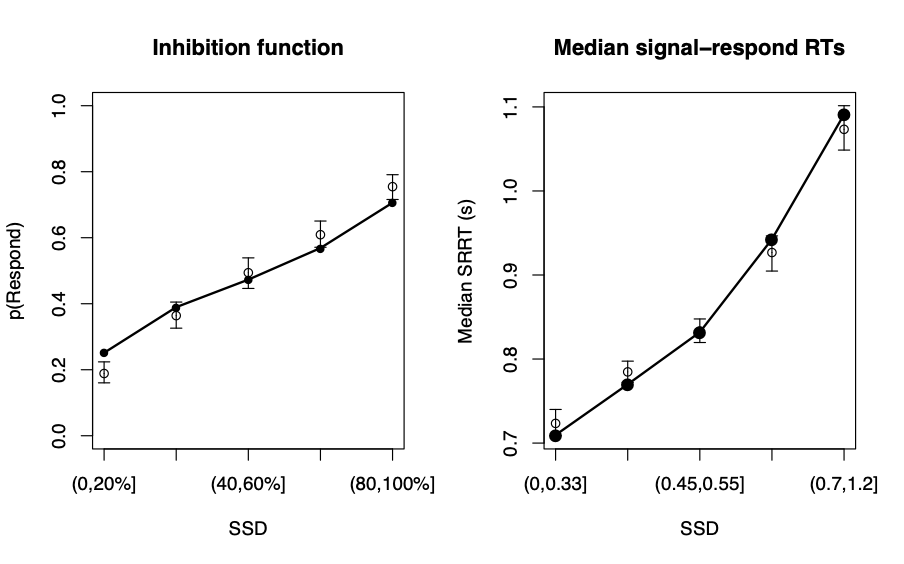


Figure A6. Observed and predicted response proportions (left panel) and median signal-response RT (SRRT) as a function of SSDs (right panel) for the sham condition. The black dots denote the observed data and the circles the median of the 500 samples of the posterior predictive distribution. The error bars denote 95% credible intervals. The SSD categories in the left panel were created by computing the percentiles of the individual SSD distributions and then averaging across individuals. In the right panel, the percentiles were calculated based on pooled individual SSDs.
